# Human Dendritic Cells: Their Heterogeneity and Clinical Application Potential in Cancer Immunotherapy

**DOI:** 10.3389/fimmu.2018.03176

**Published:** 2019-01-21

**Authors:** Thiago A. Patente, Mariana P. Pinho, Aline A. Oliveira, Gabriela C. M. Evangelista, Patrícia C. Bergami-Santos, José A. M. Barbuto

**Affiliations:** ^1^Laboratory of Tumor Immunology, Department of Immunology, Institute of Biomedical Sciences, University of São Paulo, São Paulo, Brazil; ^2^Discipline of Molecular Medicine, Department of Medicine, Faculty of Medicine, University of São Paulo, São Paulo, Brazil

**Keywords:** human dendritic cells, DC, monocyte-derived dendritic cells, mo-DC, cancer vaccines, cancer combination therapies

## Abstract

Dendritic cells (DC) are professional antigen presenting cells, uniquely able to induce naïve T cell activation and effector differentiation. They are, likewise, involved in the induction and maintenance of immune tolerance in homeostatic conditions. Their phenotypic and functional heterogeneity points to their great plasticity and ability to modulate, according to their microenvironment, the acquired immune response and, at the same time, makes their precise classification complex and frequently subject to reviews and improvement. This review will present general aspects of the DC physiology and classification and will address their potential and actual uses in the management of human disease, more specifically cancer, as therapeutic and monitoring tools. New combination treatments with the participation of DC will be also discussed.

## Introduction

Identified in mouse spleen for their peculiar shape and capacity to activate naïve lymphocytes (1–3), dendritic cells (DC) are considered the most efficient antigen presenting cells (APC) (3, 4), uniquely able to initiate, coordinate, and regulate adaptive immune responses. Though their ability to capture, process and present antigens is considered their main characteristic, their phenotypic heterogeneity is striking and very different consequences can come from their action. This review will present an overview of the main subpopulations of human DC described and will focus on their potential translational use.

## Overview of Dendritic Cells in the Immune System Physiology

Human DC are identified by their high expression of major histocompatibility complex (MHC) class II molecules (MHC-II) and of CD11c, both of which are found on other cells, like lymphocytes, monocytes and macrophages (5–12). DC express many other molecules which allow their classification into various subtypes (Table 1). Although some of the DC subtypes were originally described as macrophages, DC and macrophages have distinct characteristics (13–15) and ontogeny, so that, currently, little doubt remains that they belong to distinct lineages (16–24).

**Table 1:** Main surface markers of human and mouse DC subtypes.

**DC subtype**	**Human**	**Mouse**
cDC1	CD141/CLEC9A/XCR1	CD8a/CD103/XCR1
cDC2	CD1c/CD172a	CD11b/CD172a
pDC	CD123/CD303/CD304	B220/SiglecH
LC	Langerin/CD1a	Langerin/CD24

DC can be found in practically all tissues, where they detect homeostatic imbalances and process antigens for presentation to T cells, establishing a link between innate and adaptive immune responses. Furthermore, DC can secrete cytokines and growth factors (25) that modify ongoing immune responses, and are influenced by their interactions with other immune cells, like natural killer (26–28) and innate lymphoid cells (ILCs) (29).

**Figure 1:**
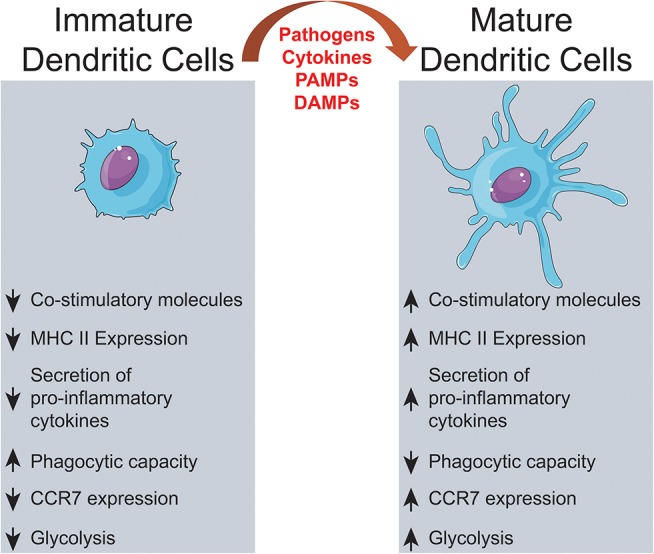
Dendritic cells activation. Extracellular signals, such as PAMPs or DAMPs, trigger alterations on immature DCs culminating on significant changes on surface proteins, intracellular pathways and metabolic activity.

DC are found in two different functional states, “mature” and “immature”. These are distinguished by many features, but the ability to activate antigen-specific naïve T cells in secondary lymphoid organs is the hallmark of mature DC (30–32). DC maturation is triggered by tissue homeostasis disturbances, detected by the recognition of pathogen-associated molecular patterns (PAMP) or damage-associated molecular patterns (DAMP) (33, 34) (Figure 1). Maturation turns on metabolic, cellular, and gene transcription programs allowing DC to migrate from peripheral tissues to T-dependent areas in secondary lymphoid organs, where T lymphocyte-activating antigen presentation may occur (35–40).

**Figure 2:**
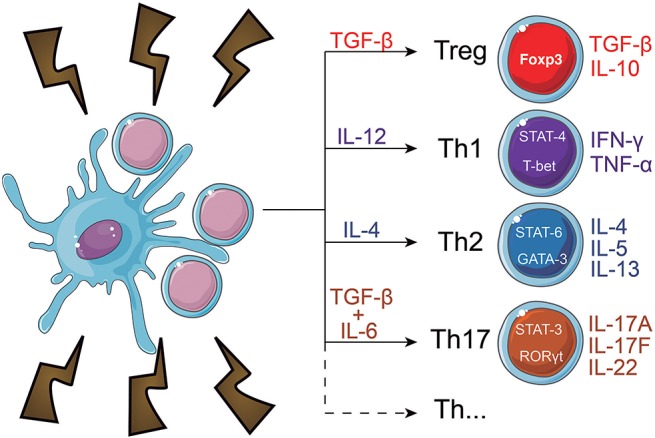
CD4+ T cell fate induced by dendritic cells. When in contact with DC, naïve CD4+ T cells can differentiate into a number of subtypes. Among them, are regulatory T cells (Treg) and T helper (Th) subsets, which include Th1, Th2, and Th17 cells. Each subtype expresses different transcription factors, which regulate the function and cytokine secretion pattern of the cells. The T cell fate decision is a complex phenomenon that heavily depends on the interaction of DC with the T cells and the cytokines present in the microenvironment.

During maturation, DC lose adhesive structures, reorganize the cytoskeleton and increase their motility (41). DC maturation also leads to a decrease in their endocytic activity but increased expression of MHC-II and co-stimulatory molecules (42–44). Mature DC express higher levels of the chemokine receptor, CCR7 (45–48) and secrete cytokines, essential for T-cell activation (42, 49–52). Thus, the interaction between mature DC and antigen-specific T cells is the trigger of antigen-specific immune responses (53, 54). When interacting with CD4+ T cells, DC may induce their differentiation into different T helper (Th) subsets (52) such as Th1 (55–60), Th2 (56, 57, 61, 62), Th17 (63–65), or other CD4+ T cell subtypes (66) (Figure 2). T cell differentiation in each subtype is a complex phenomenon, that can be influenced by the cytokines in the DC tissue of origin (67), their maturation state (42) and cause of tissue imbalance (68). However, this process is not completely elucidated, as, for example, the source of IL-4 during Th2 responses, which is discussed extensively elsewhere (69).

DC present a unique characteristic: the ability to perform cross-presentation (70–74). This phenomenon was described in 1976, by Bevan (75) and is defined as the presentation, in the context of class I MHC molecules (MHC-I), of antigens captured from the extracellular milieu. This feature allows DC to trigger responses against intracellular antigens from other cell types, thus providing means for the system to deal with threats that avoid professional APC (70, 76, 77) and, even, to prime CD8+ lymphocytes in the absence of CD4+ T cells (78, 79). Cross-presentation is involved also in the induction of tolerance to intracellular self-antigens that are not expressed by APC and, then, called, cross-tolerance (80, 81).

Before receiving maturation stimuli, DC are said to be in an “immature state.” Immature DC are poor inducers of naïve lymphocyte effector responses, since they have low surface expression of co-stimulatory molecules, low expression of chemokine receptors, and do not release immunostimulatory cytokines (44, 82). These “immature” cells, though, are very efficient in antigen capture due to their high endocytic capacity, via receptor-mediated endocytosis, including lectin- (83–85); Toll-like- (86–88), FC- and complement receptors (89) and macropinocytosis (84). Thus, immature DC act, indeed, as sentinels against invading pathogens (32, 90), but also as tissue scavengers, capturing apoptotic and necrotic cells (91).

This latter feature confers to immature DC an essential role in the induction and maintenance of immune tolerance (31, 92–95). Apoptotic cells that arise in consequence of natural tissue turnover (96, 97) are internalized by DC but do not induce their maturation (31, 98–100). Thus, their antigens are presented to T cells without the activating co-stimulatory signals that a mature DC would deliver, resulting in T cell apoptosis (80, 101), anergy (102, 103) or development into regulatory T cells (104, 105).

These “tolerogenic DC” express less co-stimulatory molecules and proinflammatory cytokines, but upregulate the expression of inhibitory molecules (like PD-L1 and CTLA-4), secrete anti-inflammatory cytokines (IL-10, for example) (102, 106–108) and are essential to prevent responses against healthy tissues (30, 31, 109–112). However, in some contexts, immature DC can be harmful to the body. It is known that DC that are unable to induce lymphocyte effector responses may contribute to the immune system's failure to fight infections (113, 114) or tumors (115–120). In these situations, DC, even after recognition of pathogens or other changes in microenvironment, fail to increase the co-stimulatory molecules required to activate T cells, thus allowing the disease to “escape” immune control.

Although many factors are recognized as contributing to drive DC maturation (100, 121, 122), the full set of such factors is not precisely defined, but involves a long series of transcriptional adaptations (119, 121, 123–125). The complexity and heterogeneity of these adaptations allows DC to translate effectively (most of the times) the pattern of homeostatic disturbance to interacting T lymphocytes, thus establishing DC as the main connector between innate and acquired mechanisms of immunity (43, 126).

## Human Dendritic Cell Subpopulations and Monocyte-Derived Dendritic Cells

Dendritic cells can be divided into resident lymphoid tissue DC and migratory non-lymphoid tissue DC (16). Both are heterogeneous cell populations with different subsets that can be distinguished by phenotypic markers and genetic profile. The first identification of different DC subsets arose from the observation that CD8 expression occurred on some, but not all, mouse resident splenic and thymic DCs (127). While the identification of mouse DC subpopulations is well advanced (128, 129), mostly due to tissue accessibility, the same is not true for human DC, where most studies were performed only in peripheral blood or skin, in spite of recent data characterizing DC subpopulations in human lung (130) and intestine (131).

**Figure 3:**
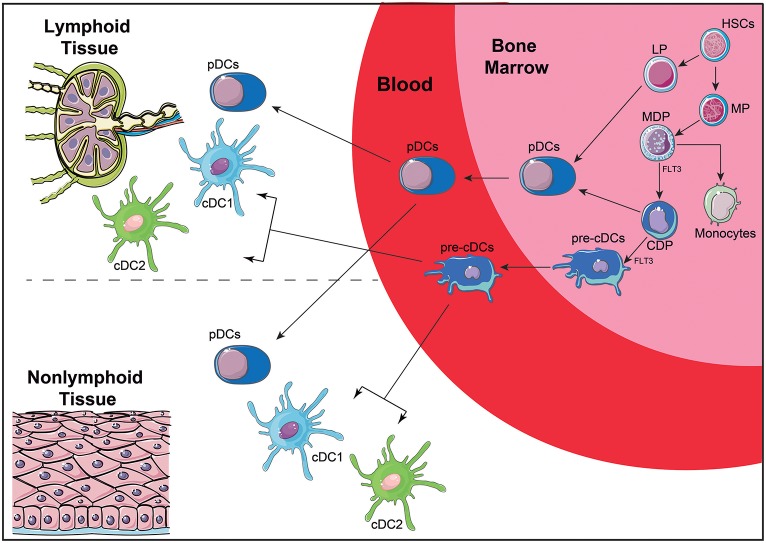
Simplified scheme of DC ontogeny. DC arise from HSC that give rise to MP and LP. MP are further differentiated into MDP that can differentiate into CDP and monocytes. CDP differentiate further into pDC or pre-cDC. LP can also give rise to pDC, although this ontogenic pathway is not completely elucidated. Once in the blood, pre-cDC give rise to two of the main DC subtypes: cDC1 and cDC2. Both pDC and cDC can migrate from the blood to lymphoid and non-lymphoid tissues. HSC, hematopoietic stem cell; MP, myeloid precursors; LP, lymphoid precursors; MDP, macrophage-DC precursors; CDP, common DC precursors; pre-cDC, pre-classical dendritic cells; pDC, plasmacytoid dendritic cells; cDCs, conventional dendritic cells; FLT3, Fms-Related Tyrosine Kinase 3.

Recent efforts have been addressed to understand the ontogeny and function of human DC subsets, attempting to correlate well-defined murine subpopulations with those found in human peripheral blood (16, 128, 132). DC arise from a CD34^+^ hematopoietic precursor that gives rise to myeloid (MP) and lymphoid (LP) precursors (Figure 3). MP differentiate into monocyte, macrophage and DC precursors (MDP), which will give rise to monocytes and to the common DC precursors (CDP). CDP can differentiate into plasmacytoid DC (pDC) or the preclassical DC (pre-cDC). Pre-cDC are the progenitors of the two major cDC subpopulations named cDC1 and cDC2 (14), which will be further discussed latter. Recent technologies, such as single cell RNAseq, are allowing a better characterization of DC ontogeny and the identification of DC subset precursors in peripheral blood (133), demonstrating that the commitment with a DC subset may be an early event, both in mice (134) and humans (135).

Curiously, in lymphohematopoietic tissue, such as spleen, thymus and blood, DC commitment to a subpopulation is mainly defined by ontogeny, while in non-lymphohematopoietic tissue, such as lung and skin, DC subpopulations are more influenced by signals derived from the microenvironment. This, once again, confirms that DC are a very plastic cell population that can shape its phenotype to the microenvironment and to homeostatic state of the tissue where it is located (136).

**Figure 4:**
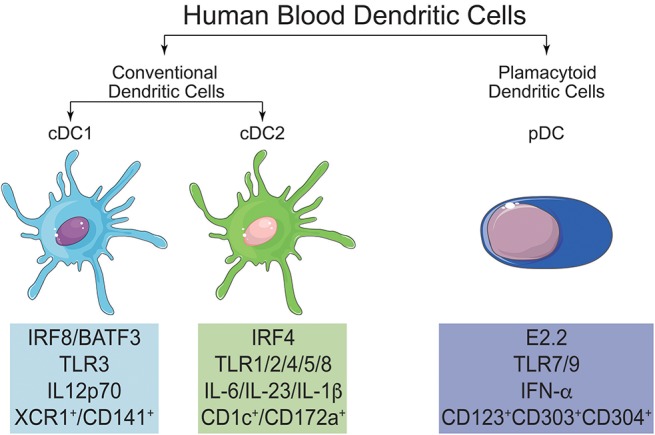
Main characteristics and differences of cDC1, cDC2, and pDC. In human blood, it is possible to find two main populations of DC, named conventional DC (cDC) and plasmacytoid DC (pDC). cDC can be further subdivided in cDC1 and cDC2. All three subtypes of DC can be differentiated by their signature transcription factors and also by the expression of specific surface markers.

In blood, DC constitute a rare cell population that can be broadly divided into two subtypes (Figure 4): CD123^+^CD11c^−^ DC, called plasmacytoid DC (pDC), and CD123^−^CD11c^+^ cells, called classical DC or myeloid DC (cDC) (25, 128, 137). Dzionek et al. (138) identified three antigens called BDCA-2, BDCA-3, and BDCA-4 (Blood Dendritic Cell Antigens), which, together with BDCA-1 (CD1c), allowed the further discrimination of human blood DC subsets. cDC can be separated into cDC1 and cDC2 (139): cDC1 are characterized by the expression of BDCA-3 (CD141) and Clec9A, while cDC2 express CD1c. BDCA-2 (CD303) and−4 (CD304), on the other hand, together with CD123, characterize pDC.

It is noteworthy that recent genomic studies, with emphasis on the subpopulations of monocytes and DC, made it possible to align CD141^+^ DC (cDC1) and CD1c^+^ (cDC2) from human peripheral blood with the mouse CD8α^+^/CD103^+^ and CD11b^+^DC, respectively (140, 141). This will allow the confirmation, or not, of the roles played by these subsets in murine immune responses also in humans.

### cDC1

The human cDC1 subpopulation is present in blood and in lymphoid and non-lymphoid tissues (142). This subpopulation is characterized by the expression of CD141, the chemokine receptor XCR1, C-type lectin CLEC9A, the cell adhesion molecule CADM1, and is the counterpart of mouse CD8α^+^/CD103^+^ cross-presenting DC subset (132, 142). cDC1 can be generated *in vitro* from CD34^+^ progenitors after 21 days of culture with fms-like tyrosine kinase 3 ligand (Flt3L) and thrombopoietin (TPO) (143) or with Flt3L and murine bone marrow stromal cell lines (144). As mentioned above, this subpopulation of DC seems to be specially adapted to perform cross-presentation, a phenomenon that is associated with the expression of the chemokine receptor XCR1 (145). The main transcription factors (TF) shown to be essential for the generation of cDC1 are the basic leucine zipper transcriptional factor ATF-like 3 (BATF3) (146) and IFN-regulatory factor 8 (IRF8) (130). In mice, besides BATF3 (147) and IRF8 (148), gene knockout models pointed out to the role of two other TF: DNA binding protein inhibitor ID2 (149) and nuclear factor interleukin-3-regulated protein (NFIL3) (150), whose participation in the generation of human cDC1 needs yet to be demonstrated.

cDC1 prime CD8^+^ T cells efficiently, what is important in anti-tumor and anti-virus immunity. However, the induction and modulation of an immune response is a very complex phenomenon that involves many cell interactions, including interactions among different DC subsets, as recently demonstrated in mice infected with modified vaccinia virus Ankara (MVA) (151). In this model, activated CD8^+^ T cells recruit both pDC (via CCL3/CCL4) and cDC1 (via XCL1); type I interferons, (IFN-I) produced by pDC, act on cDC1 optimizing their maturation, costimulatory capacity and ability to cross-present viral antigens, thus leading to an effective anti-virus response. cDC1 were also shown to be important for the antitumor activity induced by heat-inactivated MVA in murine melanoma and colon cancer models (152). Furthermore, both in mice and humans, cDC1 are found sparsely distributed along tumor margins (competing with tumor associated macrophages–TAM-for tumor antigens?) and their presence was important for the success of adoptively transferred cytotoxic T cells (CTL) (153) and for the delivery of tumor antigens to the draining lymph nodes, in a CCR7 dependent manner (154).

### cDC2

cDC2 constitute a heterogeneous subset of DC that can be found in blood, lymphoid and non-lymphoid tissue (16, 142). SIRPα (CD172a) is expressed by cDC2 (both in humans and mice) (130) and, along with CD1c (humans) and CD11b (mice), characterizes this subpopulation (25, 132). Coherently with its heterogeneity, other markers are expressed by cDC2, according to their localization, as for example, CD1a in dermal and CD103 in gut cDC2 (25, 141). Like cDC1, cDC2 can also be differentiated from CD34^+^ progenitors, after 21 days of culture with Flt3L and TPO (143) or with Flt3L and murine bone marrow stromal cell lines (144). More than one transcription factor is involved in cDC2 differentiation and IRF4 seems to be the master transcription factor (155), but other transcription factors are required. In mice, PU.1 (156), RelB (157) and recombining binding protein suppressor of hairless (RBP/J) (158) were shown to be associated with the differentiation of cDC2, and in humans, IRF8 (159).

Again, in accordance with their heterogeneity and innate plasticity (132), cDC2 have been show to induce Th1, Th2, and Th17 responses (160, 161). The puzzling heterogeneity of these cells is further illustrated by the recent description of two novel DC subtypes within the CD1c^+^ subpopulation: DC2 and DC3. These two subpopulations diverged by the expression of CD32B and CD163/CD36. Functional experiments showed that both these cDC2 subtypes were potent stimulators of naïve T cell proliferation, but show a different pattern of cytokine secretion after stimulation with a series of toll like receptors (TLR) agonists (162).

In the immune system physiology, cDC2 seem to have many, but frequently, regulatory roles. These cells have been described as potent inducers of regulatory T cells in intestine (141), and as responsible for maintaining tolerance in the liver (163). Also, cDC2 have been described as the only DC subset able to produce retinoic acid upon stimulation with vitamin D3, thus stimulating CD4^+^ naïve T cells to express gut-homing molecules and to produce Th2 cytokines (164).

### Plasmacytoid DC (pDC)

The pDC subpopulation is a subset of DC distinct from cDC, that arises directly from the CDP (while cDC arise from pre-DC precursor) (14). These cells are characterized by the secretion of high levels of IFN-α/β upon TLR7/9 stimulation, and are extremely important in viral infections (165). This subset of DC is phenotypically distinct in mice and humans. In mice, it is characterized as CD11c^int^CD11b^−^B220^+^SiglecH^+^CD317^+^ while in humans it is characterized by the absence of expression of CD11c and the expression of CD123, CD303, and CD304 (25, 128, 132). In terms of transcription factors, on the other hand, both mouse and human pDC seem to depend on the same master transcription factor, E2.2 (25, 132, 166).

Since the secretion of IFN-α/β is the main feature of pDC, their association with viral infections is not surprising. The secretion of IFN-α/β by pDCs can be a consequence of direct viral infection [like in HIV infection, where the virus infects pDC via CD4, CCR5 and CXCR4 (167)], or from external stimuli. Indeed, human pDC were shown to secrete high levels of IFN-α/β in *Aspergilus fumigatus* infection in a Dectin-2-dependent manner (168).

In keeping with the other DC subpopulations heterogeneity, human pDC may be subdivided into two subpopulations, distinguished by the expression of CD2 (169). Both pDC subsets secrete IFN-α/β efficiently, but only the CD2^hi^ subset secretes IL-12p40 and induces CD4^+^ T cell proliferation. These data, however, may be in need of a second look. As mentioned before, single cell RNAseq analysis is providing new data and allowing better characterization of DC subpopulations. When this approach was used to study pDC subpopulations, a “contaminant” putative precursor of cDC (pre-cDC), characterized as CD123^+^CD33^+^CD303^+^CD304^+^CD2^+^, was identified. When these putative pre-cDC and “pure” pDC populations (characterized by the absence of CD2 and CD33 expression) were separated and stimulated, only pre-cDC were able to induce CD4^+^ T cell proliferation and secrete IL-12p40 (135). This raises the possibility that many of the observed attributes of pDC, such as their ability to induce Th1 responses (170), to perform cross-presentation (171), to exhibit naïve T cell allostimulatory capacity (169) and expression of co-stimulatory (172) molecules might reflect the activity of this contaminating pre-cDC population.

Puzzling, as these data may seem, they illustrate quite well the plasticity of the cells “clustered” under the name of DC. They further suggest that attempts to classify strictly these cells may lead to more confusion than it is necessary to understand their role in responding to microenvironmental challenges, in shaping immune response patterns in the body and, eventually, in driving the immune response toward therapeutic goals in humans.

### Monocyte-Derived DC (mo-DC)

Much of the knowledge acquired in the past years about human DC biology was possible due to the methodology of *in vitro* deriving DC from CD34^+^ precursors (stimulated with GM-CSF and TNF-α) (173) or from monocytes (stimulated with GM-CSF and IL-4) (174). Like cDC2, mo-DC depend on IRF4 for their differentiation (175). However, they do not seem to be an equivalent population, since they arise from different precursors (14).

In mice, the precursors used for *in vitro* generation of DC are extracted from the bone marrow. In the presence of GM-CSF, these precursors give rise to large number of cells that resemble tissue DC and are called bone marrow-derived dendritic cells (BMDC) (176). Helft et al. showed that BMDC comprise a heterogeneous population expressing both CD11c and MHCII. A CD11c^+^MHCII^int^ population seems to be more closely related to macrophages (hence, called GM-Macs), while the CD11c^+^MHCII^high^ population resembles DC and is, thus, called GM-DC. Addition of IL-4 to these cultures limits, but does not eliminate, the generation of GM-Macs (177). The heterogeneity of precursors and cell populations obtained *in vitro* fuels a vivid and complex discussion about the biological relevance of these cells (178–180).

It is still unclear to which subpopulation of DC, mo-DC are more closely related, but DC ontogeny data suggest that mo-DC are similar to the inflammatory DC (132). Not surprisingly, inflammatory DC is the designation of another heterogeneous subpopulation of DC, typically CD11c^hi^MHCII^hi^. One of the first reports of inflammatory DCs described a population of DC characterized by the production of TNF and iNOS, named Tip-DCs (181). Another study identified inflammatory DC in the skin of atopic dermatitis patients and named these cells inflammatory epidermal dendritic cells (IDECs), which were characterized by the expression of CD11c, CD206, CD1a, CD11b, CD209, FcεRI (182). Recently, another inflammatory DC population was described in the synovial fluid of rheumatoid arthritis patients and in the inflammatory ascites of untreated cancer patients. In this study, inflammatory DC were characterized as CD14^+^CD1c^+^SIRPα^+^CD206^+^FcεRI^+^ and their gene signature (when compared to *in vitro* generated mo-DC, macrophages, cDC2, CD16^+^ monocytes and CD14^+^ monocytes) was more closely related to that of mo-DC, suggesting that inflammatory DC could be, indeed, the *in vivo* counterparts of mo-DC (183).

## Mo-DC as a “Window” to Immune System Evaluation in Cancer Patients

It has been known for a while that established tumors affect their microenvironment in ways that facilitate their persistence and progression. These local modifications include zones of hypoxia, altered pH, induction of angiogenesis (184), alterations of pre-mRNA splicing in surrounding cells (185) and the recruitment of cells that facilitate tumor progression, such as tumor-associated macrophages (TAM) (186), immature DC (115), myeloid-derived suppressor cells (MDSC) (187) and regulatory T cells (188). However, mechanisms to avoid immune system surveillance and tumor progression (189) are not limited to the tumor site and, today, it is recognized that individuals with cancer present also systemic modifications to that effect as well (190). As discussed before, DC are a plastic and heterogeneous population and it should be expected that, among these systemic adaptations, some affect the various DC subpopulations, including mo-DC.

### Described Alterations in mo-DC of Cancer Patients

Various publications have described phenotypic and functional alterations in mo-DC from patients with different tumors (191–193). Our group demonstrated that mo-DC from breast cancer patients are poor stimulators of allogeneic T lymphocytes proliferation but are good inducers of regulatory T cells. These characteristics were observed both in immature and mature mo-DC and the regulatory T cell bias, though decreased by blocking of TGF-β, was not completely inhibited (192). Similar phenomena were also observed in patients with CLL, whose mo-DC expressed reduced levels of important molecules involved in antigen presentation and lymphocyte activation, such as HLA-DR, CD80, CD86, CD83, and CD40, and, coherently, were less effective in inducing proliferation of both CD4^+^ and CD8^+^ T cells. Furthermore, CD4^+^ T lymphocytes co-cultivated with mo-DC from CLL patients presented reduced IFN-γ and IL-4 production, when compared to healthy donors (193). Further similar results were also observed in chronic myeloid leukemia (194), colorectal cancer (195), and cervical neoplasia (196). It is worth noting that dysfunctional and apoptosis prone mo-DC were also obtained from healthy donors, when their monocytes were exposed to tumor culture supernatants (197).

Although detected in cancer patients, the altered phenotype and functions of mo-DC could precede the emergence of the tumor and reflect an individual constitutional characteristic of the patients, which might be related or not to their disease. The follow up of cancer patients that present such alterations, however, suggests otherwise and indicate that, indeed, it is the presence of the tumor that affects the cells.

In a study of a chromophobe renal carcinoma patient, mo-DC obtained before surgery induced less allogeneic T cell proliferation and more regulatory T cells when compared to cells from healthy donors. Three months after surgery, yet, mo-DC from the patient exhibited functional properties similar to that of healthy controls, suggesting that the presence of the tumor was the cause of the biased mo-DC function in the patient (198). Another example of the transitory and, possibly, in this case, tumor-dependent functional bias of circulating cells has been described in a study with patients with obstructive jaundice. Monocytes from 53 patients with obstructive jaundice (44 due to cancer and 9 due to non-neoplastic diseases) were obtained before surgery and found unable to release H_2_O_2_ upon stimulation, but this was progressively reversed after surgery (199). Yet, in another paper we described a patient with type 2-papillary renal cell carcinoma, whose mo-DC also presented functional biases. Though after the tumor was surgically removed, the patient's mo-DC already regained some activity, their T lymphocyte-stimulating activity reached healthy controls' levels only after the patient was submitted to treatment with a dendritic cell-based cancer vaccine (200).

Altogether, these data point out to the fact that circulating monocytes may reflect systemic effects of tumors in such a manner that their functional evaluation could become an effective tool to monitor disease progression and/or response to therapy.

### Alterations in Circulating Subpopulations of DC in Cancer Patients

Circulating subsets of DC are also affected in cancer patients. Diminished numbers of total DC have been observed in melanoma patients; this was more intense in stage IV patients and, though it was more pronounced in the pDCs, it also occurred among cDC (201). In breast cancer patients, reductions in total circulating DC and in DC IL-12 production was also described. However, in these patients cDC were the culprit and not pDC (117). Circulating DC isolated from patients with CLL showed decreased expression of co-stimulatory molecules, lower ability to stimulate allogeneic T lymphocytes and did not secrete IL-12, but retained the ability to secrete IL-10 (202). A recent publication, evaluating the effects of different TLR-L in cDC1, cDC2, pDC, and monocytes from breast cancer patients showed that, upon stimulation with IFN-α, cDC2 and non-classical monocytes (CD14^−^CD16^+^) exhibited reduced secretion of TNF-α (203).

These observations point out to systemic effects induced by tumors upon the immune-hematopoietic system and suggest that circulating cells are influenced and, possibly functionally handicapped to fight the tumor, even before actually infiltrating the tumor mass. These phenomena, added to our view and understanding of tumor biology, should allow the design of improved therapeutic approaches, even for those that do not specifically target the immune system.

### Possible Mechanisms

It is quite evident, thus, that tumors promote local and systemic alterations in immune cells and substantial efforts have been made to identify possible mechanisms of how tumors promote these alterations and, most importantly, how to correct them.

Signal Transducer and Activator of Transcription 6 (STAT6) is an important molecule, induced by IL-4, in the process of mo-DC differentiation. STAT6 is naturally inhibited by the Suppressor Of Cytokine Signaling 5 (SOCS5), which, in turn, is up regulated by phosphorylated STAT3 in monocytes. In CLL patients, IL-10 induces the phosphorylation of STAT3, thus up regulating the expression of SOCS5. As a consequence, monocytes of CLL patients have impaired phosphorylation of STAT6 and its downstream genes, blocking their differentiation and maturation into functional mo-DC (193). However, mo-DC from healthy donors differentiated in the presence of lung cancer patients' sera, showed decreased STAT3 phosphorylation (204). Although apparently contradictory, these findings might reflect a difference in the monocytes of patients and healthy donors or a difference in the moment of analysis. If monocytes from patients and healthy donors differ, it would not be surprising that they would respond differently to the same stimuli. Likewise, the moment when STAT3 phosphorylation is analyzed may show quite different results. When monocytes from healthy donors were pre-treated with IL-10 and then stimulated with IL-4, an initial increase in STAT3 phosphorylation occurred during the first 72 h, but with the increasing SOCS5 expression, STAT3 (and STAT6) phosphorylation was downregulated (193).

The STAT3 pathway is activated also by IL-6, which, like IL-10, is found in higher concentration in patients sera (205). The impaired functions of DC have been, thus, also attributed to upregulation of IL-6-induced STAT3 activity, both in animal models (206) and humans (207)- these data were recently reviewed by Kitamura et al. (208). Offering a potential solution to these hard to reconcile data is the fact that STAT3 signaling induced by IL-6 seems to be modulated by SOCS in a different way than the IL-10-induced signaling, at least in human macrophages (209).

Undeniably, the available data, though suggesting possible pathways are not enough to elucidate the complex molecular mechanisms underlying DC dysfunction in patients.

## Dendritic Cells as Therapeutic Instruments

The key concept of the cancer immunotherapy is that the manipulation of the immune system can achieve cancer control and, ideally, cure. The possibility of cancer immunotherapy was first shown by Coley, who used a mixture of bacterial toxins to treat patients with inoperable sarcomas (210). Since then, many studies have shown clinical benefit when using general immune system activators, such as bacterial products (211) and TLR agonists (212). The antitumor activity of these approaches, when it occurs, is attributed to the ability of these compounds to activate the immune system that, in turn, acquires the ability to kill tumor cells. Much of this effect was shown to be due to DC activation followed by the generation of T cell responses (213). Dendritic cells, as key activators of the adaptive immune response, would be expected to have a central role in inducing antitumor immune responses and the many functional deviations these cells show in cancer patients emphasize the relevant role they may, indeed, play in anti-tumor immune responses. In face of these data, it would be intuitive to exploit the immune activating potential of DC to induce antitumor responses in cancer patients. However, because of the difficulty of obtaining large numbers of these cells by non-invasive methods, therapeutic approaches using DC became possible only after methods for the *in vitro* generation of these cells were described (174).

### Use of Monocyte-Derived Dendritic Cells

mo-DC are able to present antigens in the context of both MHC class I (91) and class II molecules (214) and, hence, can be used to generate therapeutic cancer vaccines. When injected in humans, mo-DC can prime CD4+ and CD8+ T cells (215) and expand antigen-specific cytotoxic T cells, which can lead to regression of metastatic lesions in patients (216). Nevertheless, some argue that mo-DC, possibly due to a limited migration potential, might be insufficient to consistently induce effective immune responses *in vivo* (217). Contrastingly, Kuhn and co-workers have shown that successful therapy using immune-activating compounds was followed by the appearance of mo-DC in the draining lymph nodes of treated mice (218) and these cells were essential for the priming of CD8+ T cells and antitumor immunity (219).

**Figure 5:**
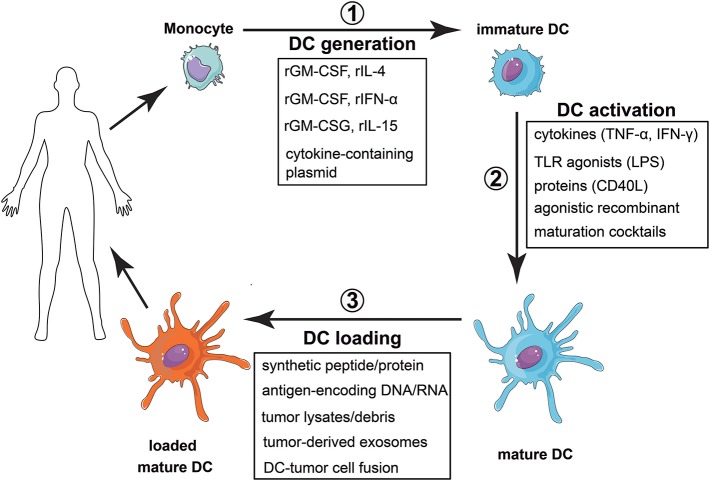
Vaccination strategy with monocyte derived dendritic cells. (1) Monocytes are obtained from peripheral blood and differentiated into dendritic cells. This differentiation can be achieved by using different recombinant cytokines, with rGMCSF + rIL-4 as the most common combination, or by transfecting monocytes with plasmids encoding the cytokines. (2) Once differentiated, DC activation can be accomplished by using different stimuli, most of them associated with tissue damage, inflammation or the presence of a pathogen. (3) The last step is to load the DC with selected or total tumor antigens. Finally, the cells are injected in the patient, expecting them to induce a tumor-specific adaptive immune response able to eradicate the tumor.

Nonetheless, to be used as therapeutic instruments, mo-DC must be properly differentiated *in vitro*, induced to mature, loaded with tumor antigens, and, finally, administered to the patient (Figure 5). It is easy, thus, to realize the challenges that face the development of mo-DC-based vaccines. What are the markers of a “properly activated” DC? What is the “proper” response to be induced? What are the relevant tumor antigens? What is the best pathway for these cells to reach secondary lymphoid organs, where they should encounter tumor-specific T lymphocytes? Not surprisingly, each of the aforementioned steps diverges among the various clinical reported protocols, adding much complexity to the evaluation of the approach, but also a possible explanation for the large diversity in the reported efficiencies of such treatments.

To differentiate monocytes into dendritic cells, the cytokines IL-4 and GM-CSF are classically used (174). Most approaches use this protocol to obtain mo-DC, but other ways to differentiate monocytes into dendritic cells have been described and tested. mo-DC differentiated in the presence of GM-CSF and IFN-α, for example, secrete large amounts of pro-inflammatory cytokines, induce a IL-12p70-independent Th1 response (220) and have given rise to cancer-specific CD8 responses, in phase I/II clinical trials (221). mo-DC differentiated in the presence of GM-CSF and IL-15, on the other hand, were better inducers of Th17 responses (222).

The lengthy culture time to achieve the differentiation of mo-DC (usually 5–7 days) is a limitation of the wide clinical use of these protocols. Thus, alternative protocols for mo-DC differentiation were developed. Dauer et al. have shown that monocytes cultured for 48-hours with IL-4 and GM-CSF already have characteristics of immature DC (223) and these, so called FastDC, prime tumor-antigen specific CD8 T cells as efficiently as conventional mo-DC (224). Another strategy is the transduction of monocytes with plasmids containing the genes of the cytokines, which, constitutively expressed, will lead to their differentiation into DC (225). The FDA-approved cancer vaccine, Sipuleucel-T (PROVENGE®) uses a similar approach for mo-DC generation, in a protocol that only requires 3 days for manufacturing (226). This vaccine is approved for castration-resistant prostate cancer and consists of autologous PBMC incubated with a fusion protein containing both GM-CSF and PAP, a prostate-specific cancer-associated antigen (227).

The second step in vaccines generation consists of mo-DC activation, since differentiation generates immature cells. The maturation stimulus can come from a variety of molecules, including cytokines (TNF-α, IFN-γ), TLR agonists (LPS), agonistic recombinant proteins (CD40L) or maturation cocktails (228). However, the best conditions for mo-DC activation are still unclear. Activation with TNF-α, for example, has been implicated in the induction of mo-DC with impaired ability to secrete pro-inflammatory cytokines, which could even protect mice from autoimmunity (229). On the other hand, combinations of TLR agonists synergize to promote Th1 responses (230). Vopenkova et al. made a direct *in vitro* comparison of different maturation stimuli to induce tumor-specific T cells, showing that the highest response was achieved with the combination of IFN-γ and LPS (231). However, clinical effectiveness comparisons of different mo-DC formulations are still lacking.

Next, mo-DC need to be loaded with tumor antigens. For this, bulk tumor products or selected tumor antigens have been used. Tumor associated antigens (TAAs), recognized by T cells, are found in several tumors (232). Immunodominant synthetic peptides derived from TAAs have been tested and were able to induce clinical and immunological responses of the vaccinated patients (233). Also DNA molecules encoding TAA genes can be employed to load mo-DC, in which case, viral vectors, intrinsically able to activate DC (234), bring further advantage. It is noteworthy that, for all these methods, there is no need of tumor samples from the patient, which may be scarce. However, the use of single antigens has its drawback. Due to the cellular heterogeneity of tumors, they can escape from the immune response generated by the vaccine, through the selection of cells that do not express the immunizing antigens (235). Strategies that involve the induction of a poly-antigenic response can be used to avoid this resistance, especially in melanoma, where this effect is frequently observed. Bulk tumor products may be used as a broad source of tumor antigens.

In addition to tumor lysates, living tumor cells, necrotic debris, apoptotic bodies and tumor-derived exosomes have been used (236). The type of antigenic source used, however, can interfere with the type of immune response obtained and it is impossible, today, to predict which would be the most appropriate antigenic source. For example, in mice, dendritic cells loaded with apoptotic tumor cells were show to induce better responses than tumor lysates, peptides or RNA (237), a finding that contradicts the many data showing that apoptotic cells captured by DC constitute a mechanism of immune tolerance induction.

Although several protocols of vaccination with mo-DC have been tested in clinical trials, only a few obtained relevant clinical responses, and most of them failed to reach the expected results (238). The lack of success in these approaches could be attributed to the functional alterations found in cancer patients mo-DC (239). The use of allogeneic mo-DC obtained from healthy histocompatible donors would be a strategy to bypass this problem, although limited by the need of a MHC-matched donor. Another approach is the use of dendritic cell-tumor cell hybrids. These fused cells express MHC molecules from both tumor and DC origin, forsaking the need of a MHC-matched donor to generate the mo-DC (240). They are also superior than the mixture of these cells, induce antitumor responses and clinical response in patients with advanced metastatic tumors (241). Regardless of the strategy, however, clinical responses to mo-DC-based vaccines are still beyond the desired. This suggests that it may be not enough to have an efficient antigen presentation to induce tumor regression, once it is established. Other compromises between the tumor and the immune system might still prevent an effective tumor-clearing immune response requiring the design of new approaches and, very likely, the combined targeting of different immunological pathways.

### Targeting DC Subsets *in vivo*

More recently, a new modality of DC-based immunotherapy strategy is under development. With the better DC subsets characterization and the identification of specific surface markers for these subsets, it became possible to design strategies to deliver different molecules or “packages” to these cells *in vivo* (242). This would allow the selective delivery of antigens and/or immunostimulatory molecules to defined cell subtypes *in vivo*, preventing the costly and laborious *ex vivo* mo-DC generation.

Among the most studied DC-targeting antibodies are those specific for DEC205, CLEC9A, and CLEC12A. These C-type lectin receptors are expressed, in mice, by cDC1 and, the last two, also by pDC (243). Due to their cross-presentation ability, targeting to cDC1 seems to be a reasonable choice, which would favor a higher CD8+ T cell response.

Indeed, experimental settings targeting these molecules were able to induce T cell responses (244, 245) and regression of metastatic melanoma in mice (246). Interesting and well designed as this strategy may be, in humans this strategy is still restricted to *in vitro* studies (247) and awaits, urgently, translational research.

## Strategies to Improve the Clinical Effectiveness of mo-DC-Based Therapies

Before specifically addressing the many current pathways for the improved translation of our knowledge of DC biology into clinical applications, it is worth mentioning that, though most of this effort is concentrated into the use of these cells to induce effector immune responses, it is only a matter of time till it becomes feasible to delineate DC-based strategies to treat conditions where the immune system went rogue and is causing autoimmunity, or where medical interventions require the limitation of immune responses, like organ transplantations.

That said, let us consider the strategies that may lead to enhanced immunogenic effects of mo-DC-based treatments.

### Approaches for the Improvement of DC-Based Treatments

Since mo-DC show deviant phenotypes in cancer (192) and are susceptible to negative modulation by different drugs, for example STAT5 inhibitors (248), the converse is also true and various approaches are under development to achieve the generation of “better” mo-DC.

The chemokine CXCL-4 is a powerful chemoattractant to monocytes and an important immunoregulator that has been shown to enhance the expression of MHC, CD86, and CD83 molecules by mo-DC of healthy donors, leading to more efficient antigen presentation, induction of CD4^+^ and CD8^+^ T cells proliferation and production of IFN-γ (249).

As mentioned before, IL-6 through the activation of STAT3 interferes with proper DC maturation and, indeed, in patients with colorectal cancer has been associated with poor CD4^+^ T cells responses (207). Coherently, a phase-I study in ovarian cancer patients showed that, combined with chemotherapy, IL-6 blockade was safe and induced a series of positive modifications in immune parameters of the treated patients, including increases in IL-12, IL-1β, TNF-α, and IFN-γ secretion (250).

Besides targeting the negative regulators of DC activation, it is possible to overcome this phenomenon by changing the activating signals delivered to these cells. Following this line of research, a cocktail of inflammatory cytokines (TNF-α, IL-1β, poly I:C, IFN-α, and IFN-γ) has been tested for mo-DC maturation and was shown to increase their IL-12 production and their ability to prime melanoma-antigens-specific T cells *in vitro* (251). This mo-DC activating cocktail, in a vaccination study of 22 recurrent glioma patients, was associated with increases in serum type 1 cytokines and chemokines, tumor-associated antigens-specific T cell responses and clinical benefit in 9 patients (252).

Another approach is based on the use of adjuvants to boost the immune response. Among these, GM-CSF used in vaccines as GVAX (253) and STINGVAX (254) and, even TLR agonists (255), may be more effective for cell maturation. Other adjuvants could be listed, as for example, aluminum salts (256) (an inflammasome activator), and montanide (257) (an equivalent to incomplete Freund's adjuvant). Those adjuvants may boost responses due to physical effects upon antigens and cells, but also enhance DC activation. Nonetheless, the consideration of such a heterogeneous group of substances is enough to realize that adjuvant research is a rich field that may broaden the applicability and enhance the effectiveness of DC-based vaccination (258).

A different pathway to improve the effectiveness of DC-based therapy focuses on the selection of the immunizing antigens. In cancer, the mapping of a patient's set of neoantigens and use thereof would represent the epitome of personalized medicine. Though very tempting, this approach would still have its drawbacks, a significant one being the fact that not all tumors express immunogenic neoantigens (259), not to mention the cost that such strategy would impose on any health care system. Nevertheless, its feasibility and efficacy has already been demonstrated in an elegant study (260) where personalized vaccines were prepared for 6 melanoma patients. Whole-exome sequencing of their tumors allowed the identification of the mutated antigens from which a set of peptides was selected and synthesized so that they would be presented in the context of MHC-I. Four patients presented complete clinical responses to the vaccine alone and the other two, who had progressive disease after the vaccination, experienced complete responses after treatment with anti-PD-1. Curiously, in spite of the selection of MHC-I selective peptides, both CD4+ and CD8+ antigen-specific T cells were stimulated, with a predominance of CD4+ T cell responses. This observation illustrates very well how much “real life” immune responses still differ from our predictions.

Another ingenious strategy bypasses many of the known hurdles to exploit the immunogenic potential of DC. This approach aims to deliver RNA-containing nanoparticles systemically, which due to their lipid composition would be preferentially captured by DC and, then, release the RNA encoding the selected antigen(s) to be synthesized and presented. In a murine model, this approach lead, indeed, to DC maturation, IFN-α production and strong antigen-specific immune responses, which were effective in a series of tumor models (261). Accordingly, this strategy is under investigation in a clinical trial (NCT02410733) for patients with advanced melanoma.

### Combination Treatments Including mo-DC

Chemotherapy and radiotherapy, together with surgery, still remain as the main pillars of cancer therapy. Since chemotherapy in general was formerly considered immunosuppressive, little attention was given to the fact that this is not always true. Indeed, some drugs might potentiate the anti-tumor immune response, by inducing the now recognized “immunogenic cell death” (262, 263). However, due to the frequently observed cancer patients' DC dysfunctions, the simple immunogenic death may not be enough to disrupt the tumor-favoring status of the immune response in patients. To achieve that, active immune interventions may be necessary to take advantage of the phenomenon. Indeed, a series of studies, both experimental and in humans, has been addressing this issue with promising results (264–266).

Radiation may also favor the induction of anti-tumor immune responses and, as with chemotherapy, there are plenty of data indicating a beneficial effect of its combination with cancer vaccines or other immune-stimulating strategies in different settings, including hepatocellular carcinoma (267), prostate cancer (268), lymphoma (269), and glioblastoma (270). Currently, the potential of such combinations are under scrutiny in a series of clinical trials for patients with such disparate diseases as anal (NCT01671488), lung (NCT01579188) and pancreatic cancer (NCT01072981) (271).

The disparity of the diseases mentioned at the previous paragraphs is a good indicator of the contrast between therapeutic strategies directed against the tumor cell and those targeting the immune system. Those that aim at the tumor cell will differ significantly from one tumor to the other, since each tumor has its own set of genetic changes and will respond differently to a given treatment. On the other hand, strategies that target the immune system, though still dealing with a very complex set of interactions, will face, very frequently, standard responses of the immune system to the perturbations caused by the presence of the tumor, regardless of the tumor's set of genetic mutations.

Actually, the realization of this scenario and the better understanding of the immune system and its interactions with tumors opened the way to a very attractive and successful approach for cancer immunotherapy: instead of targeting directly the tumor, one could target the immune regulatory mechanisms that allow a frequently immunogenic tumor to grow in an otherwise immunocompetent host. With this, the “checkpoint inhibitors era” started and achieved unprecedented good clinical results (272), leading to this 2018's award of the Nobel Prize in Medicine for James Allison and Tasuku Honjo for their work in this area.

However, after the initial excitement and even after the inclusion of other checkpoint inhibitors among the available armamentarium against cancer, it is necessary to appreciate that not all patients will respond to this approach, since it needs an existing response, kept in check and “waiting” to be released by the treatment. On the other hand, it is quite possible that the frequently unsatisfactory response to cancer vaccines is caused by the pre-existence or vaccine-induced activation of these same regulatory circuits. Hence, a coherent path to achieve better clinical results would be the combination of both immune modulating strategies. Indeed, experimental (273) and clinical data (274) suggest that this may be true. In the aforementioned clinical study, patients with advanced melanoma were treated with a combination of MART-1-peptide pulsed-DC and anti-CTLA-4 and the results indicated that the combination might be, indeed, more effective than either approach alone. Likewise, also in the PD-1/PD-L1-PD-L2 pathway (275, 276) the combination of DC vaccination with checkpoint inhibition may offer, at least theoretical, advantages.

A different set of combination treatments has been targeting immune modulatory enzymes. The enzyme indoleamine 2,3-dioxygenase (IDO) catalyzes the degradation of tryptophan contributing to tolerance induction by favoring regulatory T cell differentiation and reducing DC activity (277). IDO expression by DC is induced by inflammatory stimuli (278), but also by CTLA-4 and PD-1 (279). Accordingly, IDO inhibition has shown positive effects in murine models of pancreatic cancer (280) and a study combining IDO inhibitors with DC vaccines for breast cancer patients has completed recruitment (NCT01042535). Similarly, an inhibitor of BCR-ABL, SRC, c-KIT, PDGFR, and ephrin tyrosine kinases has shown synergistic effects with a DC vaccine in a mouse melanoma model (281) and this combination is the object of ongoing clinical trials in patients with melanoma (NCT01876212) and metastatic renal cells carcinoma (NCT02432846 phase II e NCT01582672 phase III). Arginase-1, an enzyme that regulates cell proliferation and is constitutively expressed by myeloid-derived suppressor cells (MDSC) (282) and cyclooxygenase-2 (COX2), are other two enzyme whose inhibition might have positive interactions with immunotherapeutic approaches, including those that exploit DC.

## Concluding Remarks

Dendritic cells have a central role in the immune system homeostasis and are directly involved in defining the patterns of response the system develops when facing an antigenic challenge. Their normal function warrants protection against infections, possibly cancer, but also against autoimmunity and hypersensitivity reactions. The more is uncovered of the mechanisms that drive these cells to modulate the response in one way or another, the more tools will be available to direct the immune system to desired therapeutic outcomes. Today, much of the efforts and clinical results are focused into harnessing these cells to induce effector responses, mainly, but not only, in cancer. With the advancement of the understanding of their physiology and regulatory pathways, it is possible to predict their effective use in such opposing conditions as cancer and diabetes, with less untoward and more durable effects.

## Author Contributions

TP, MP, AO, and GE reviewed the literature and wrote the manuscript. PB-S revised the literature and the manuscript and JB designed, wrote and revised the manuscript.

### Conflict of Interest Statement

The authors declare that the research was conducted in the absence of any commercial or financial relationships that could be construed as a potential conflict of interest.
